# Intermittent versus continuous drought: chlorophyll *a* fluorescence reveals photosystem resilience in tomato

**DOI:** 10.3389/fpls.2025.1699777

**Published:** 2025-11-24

**Authors:** Jesús D. Peco, Ana Centeno, Rubén Moratiel, Jaime Villena, Jesús A. López-Perales, Marta M. Moreno, David Pérez–López

**Affiliations:** 1 Departamento de Producción Vegetal y Tecnología Agraria, ETSIA–Universidad de Castilla–La Mancha, Ciudad Real, Spain; 2 Departamento de Producción Agraria, CEIGRAM–Universidad Politécnica de Madrid, Madrid, Spain; 3 Instituto Botánico, Universidad de Castilla-La Mancha, Albacete, Spain

**Keywords:** *Solanum lycopersicum*, drought resilience, water stress, OJIP profile, photosystem II, chlorophyll a fluorescence, stress integral, Mediterranean landraces

## Abstract

**Introduction:**

Recurrent drought threatens Mediterranean tomato yields, yet how the time-scale of drought shapes photochemical tolerance remains unclear.

**Methods:**

We evaluated six genotypes, three commercial cultivars (‘Sintonía’, ‘Marejada’, ‘Valenciano’) and three Mediterranean landraces (‘82’, ‘264’, ‘260’, under greenhouse conditions. Plants received either two short pulses (WS1) or a single prolonged drought (WS2). We tracked stem water potential (Ψ_stem_) and computed a stress integral (SI), and measured gas exchange, leaf chlorophyll, and chlorophyll a fluorescence (OJIP test) across key time points and after rewatering.

**Results:**

The first WS1 pulse transiently increased performance index (PI_ABS_) and electron‑transport efficiencies (Ψ_E0_, ϕ_E0_) by 20–40 % in four cultivars. Photosynthesis declined by –70 to –80 % but recovered within three days of irrigation. ‘Sintonía’ showed early increases in dissipation (ϕ_D0_) and fluxes (ABS/CS_0_, DI_0_/CS_0_), while maximum quantum yield (ϕ_P0_) remained unchanged across genotype. Sustained WS2, however, reduced PI_ABS_ and ϕ_P0_ by –18 to –50 %, increased ϕ_D0_, ABS/CS_0_ and DI_0_/CS_0_ by 30–60 % in all except ‘260’, whose OJIP profile remained stable. Photosynthesis dropped near zero but recovered in five genotypes; ‘264’ recovered only 50 %, showing irreversible damage. Chlorophyll content stayed constant, so shifts were pigment‑independent.

**Discussion:**

Findings support a three-stage resilience model: (i) reversible photoprotective adjustment to short severe drought; (ii) cumulative photochemical damage under sustained deficit; and (iii) genotype-dependent recovery.

**Conclusion:**

Combining temporal stress integrals with OJIP screening provides a robust pipeline for breeding Mediterranean tomatoes adapted to future drought, advancing knowledge of drought resilience mechanisms.

## 1 Introduction

Tomato (*Solanum lycopersicum* L.) is a crucial vegetable crop cultivated in semi–arid regions. In Spain, its primary cultivation takes place within the greenhouses of the southeastern Mediterranean regions, where water availability poses a limiting factor for crop production (Castilla, 2007; Acebedo et al., 2022). Consequently, researching the response of this crop to water stress is imperative for enhancing irrigation efficiency and ensuring the sustainability of these agricultural systems. One solution actively pursued by the scientific community involves the search and enhancement of drought–resistant varieties capable of withstanding water stress without compromising yield (Sánchez–Rodríguez et al., 2011; Conti et al., 2019). Studying local tomato landraces, frequently grown in arid areas and repeatedly exposed to water scarcity, provides valuable insights for this purpose. These crops have developed specific characteristics through natural selection and cultivation practices unique to their regions, making them well adapted to challenging environments (Moreno et al., 2019; Villena et al., 2023). Such distinctive traits could be instrumental in developing new commercial varieties better able to withstand future drought scenarios (Sousaraei et al., 2021).

Tomato is notably sensitive to moderate drought, particularly during its flowering and fruit enlargement stages (Jangid and Dwivedi, 2016). However, a shortage of soil water availability at the onset of plant growth can significantly limit biomass production and the photosynthetic capacity of leaves (Hao et al., 2019). This limitation indirectly hinders the formation of reproductive organs and, consequently, reduces yield (Wu et al., 2021). When tomato plants face drought conditions, various parameters, including chlorophyll pigments, photosynthetic rate and photochemical efficiency, among others, are markedly affected, and the rate is often genotype–dependent (Mishra et al., 2012; Giorio et al., 2018; Liang et al., 2020). The rate at which these parameters decrease with increasing water stress can provide insights into the drought tolerance of each variety. Furthermore, understanding the recovery of photosynthesis after rehydration is crucial for comprehending the profound damaging effects of water stress and discerning potential acclimatization mechanisms in plants (Torrecillas et al., 1995; Iovieno et al., 2016; Giorio et al., 2018; Peco et al., 2023). Under mild to moderate water stress, stomatal limitations predominate over non–stomatal factors, while severe stress is characterized by non–stomatal limitation. Consequently, plants exposed to mild and moderate stress typically restore their normal values shortly after rehydration. However, under severe stress, an impairment of the photosynthetic machinery occurs, preventing the recovery of normal values upon rehydration (Flexas and Medrano, 2002). Characterizing these behaviors is pivotal in the quest for crops that exhibit greater resistance to drought (Aghaie et al., 2018).

Conventionally, various parameters have been measured in tomato plants as a method to detect several environmental stresses. Stem water potential (Ψ_stem_) has been utilized as a stress indicator in water–stressed tomato leaves (Zgallaï et al., 2006; Silva et al., 2012). However, despite providing insight into the plant’s water status, this parameter offers limited information about how the plant is physiologically and biochemically affected. On the other hand, photosynthetic parameters, such as stomatal conductance, transpiration, and net photosynthesis, contribute to our understanding of the plant’s activity when exposed to different levels of water stress (Yuan et al., 2016; Giorio et al., 2018; Hao et al., 2019; Peco et al., 2023). However, these parameters do not provide a comprehensive measure of the plant’s overall physiological impact. This is because, under water stress, there is a rapid closure of stomata, leading to a swift decrease in stomatal conductance, transpiration, and net photosynthesis (Peco et al., 2021). Although these measurements can offer an indication of the plant’s tolerance to water stress, they do not yield insights into the effects on the photosynthetic machinery, specifically the photosystems (PSII and PSI) and their photosynthetic electron transport.

Previous studies have identified chlorophyll *a* fluorescence techniques as suitable indicators of water stress, enabling the monitoring of damage to the photosynthetic apparatus, specifically the PS II and I, and the electron transport chain (Mishra et al., 2012; Sousaraei et al., 2021). The chlorophyll *a* fluorescence OJIP transient technique, characterized by the O, J, I and P steps corresponding to the redox state of PS II and I, is a non–destructive, simple, and rapid testing method (Baker and Rosenqvist, 2004). This technique has been employed to assess the impact on photosynthetic components under various environmental stresses such as drought, heat, chilling, salt or heavy metals (Baker and Rosenqvist, 2004; Zushi et al., 2012; Zushi and Matsuzoe, 2017; Chtouki et al., 2021). Among the OJIP–derived indices, the performance index on absorption basis (PI_ABS_) integrates energy absorption, trapping, electron transport and dissipation into a single parameter and is considered a reliable descriptor of photosynthetic vitality (Jedmowski and Brüggemann, 2015; Chiango et al., 2021). The maximum quantum yield (ϕ_P0_ = Fv/Fm) indicates the efficiency of PS II photochemistry, while electron transport indices such as Ψ_E0_ and ϕ_E0_ describe the probability and efficiency of electron flow beyond Q_A_ towards PS I (Zivcak et al., 2014; Ceusters et al., 2019). The coefficient ϕ_D0_ reflects the proportion of absorbed energy dissipated as heat through non–photochemical processes (Bashir et al., 2021). Additional parameters, such as ABS/CS_0_, TR_0_/CS_0_ and DI_0_/CS_0_, quantify the absorbed, trapped and dissipated energy per excited cross–section, providing complementary information on the activity and connectivity of the reaction centers (Guha et al., 2013; Swoczyna et al., 2022). Together, these indices offer insights into the physiological, biochemical and biophysical state of PSII in response to drought stress. Indeed, several studies have been conducted on OJIP transients in water–stressed tomato plants (Mishra et al., 2012; Sousaraei et al., 2021). However, information for tomato remains limited compared with other crops, and the temporal dimension of drought (intermittent versus continuous) is rarely addressed, even though drought duration may strongly influence the balance between reversible photoprotection and irreversible damage. Short, intermittent deficits are known to activate transient energy-dissipation and stomatal control mechanisms that help maintain PSII functionality, whereas sustained droughts can lead to cumulative oxidative stress and photoinhibition (Flexas and Medrano, 2002; Guha et al., 2013; Zivcak et al., 2014; Peco et al., 2023). Although such temporal effects have been suggested in other crops, they remain poorly demonstrated in tomato, a gap this study aims to address.

In this research, we evaluated six tomato genotypes, including three commercial cultivars and three Mediterranean landraces, to assess their drought tolerance. Measurements of stem water potential, chlorophyll contents, photosynthetic parameters and chlorophyll *a* fluorescence (OJIP analyses) were conducted to compare the varying capacities for drought avoidance between commercial and local accessions. This research is motivated by the understanding that distinct farming practices and an emphasis on high production, without considering potential limitations in commercial plants, may result in divergent physiological and biochemical responses compared to local plants when subjected to water stress. Moreover, the scarcity of studies integrating OJIP transients with traditional drought indicators in tomato underscores the need for such a comprehensive assessment.

## 2 Materials and methods

### 2.1 Plant material and growth conditions

Tomato local cultivars, SL–82’ (‘82’), ‘SL–264’ (‘264’) and ‘SL–260’ (‘260’), were sourced from the gene bank of the Higher Technical School of Agricultural Engineers at the University of Castilla–La Mancha located in Ciudad Real (Moreno et al., 2019; Villena et al., 2023). Three commercial cultivars, ‘Sintonía’ (‘SN’), ‘Marejada’ (‘MR’) and ‘Valenciano’ (‘VL’), commonly used in intensive Mediterranean production systems, were obtained from an agricultural company in Southern Spain (NUNHEMS company) (Marín, 2021). A detailed description of the six genotypes is provided in Peco et al. (2023).

Seeds were sown in porex trays in March under controlled light, temperature and humidity conditions until the plants reached stage 104 on the BBCH scale (fourth true leaf unfolded) (Feller et al., 1995). Once this stage was attained, seedlings were transplanted in May to 40 l pots containing a mix of sand and commercial substrate (Projar Professional, Valencia, Spain) in a 1:2 ratio, with each pot holding 7.5 kg of substrate. Plants were cultivated for two months inside a greenhouse situated in the Agricultural Research Area of the Polytechnic University of Madrid (40°26′21.8″N, 3°44′15.7″W). Inside the greenhouse, the mean daily temperature was maintained at 23.2°C, with daytime peaks reaching 29.6°C and minimum night temperatures of 14.6°C; average relative humidity was 51.8 %.

Prior to applying the water deficit treatments, plants were irrigated daily to maintain soil moisture at full water–holding capacity (WHC) for a one-month stabilization period under full irrigation to ensure uniform growth before applying the drought treatments. The experiment targeted the vegetative expansion phase, as this stage offers stable physiological conditions to examine responses to drought, minimizing confounding effects from flowering and fruiting (Guichard et al., 2005). Measurements were conducted from June to July.

The trial was arranged in a randomized complete block design with three irrigation regimes, six cultivars and four replicates per treatment, resulting in 72 pots in total (one plant per pot). The irrigation treatments were defined as follows:

- Control (C): Plants received daily watering to sustain soil moisture at WHC throughout the study.
- Water Stress 1 (WS1): Plants experienced two separate drought events. Five days after transplanting, irrigation was suspended for 10 days, followed by rewatering to WHC. The next day, a second dry period of 15 days was imposed, ending with final rehydration.
- Water Stress 2 (WS2): Plants were subjected to a single prolonged drought lasting 25 days, starting five days post–transplant. When stem water potential fell below –1.4 MPa, 100 ml of water was added to sustain severe stress conditions without killing the plants. At the end of the period, they were rehydrated to WHC.

During both WS1 and WS2, drought intensity was monitored through stem water potential (Ψ_stem_), which provides an integrative measure of the plant’s water status. Irrigation was completely suspended at the onset of each stress period and maintained until Ψ_stem_ reached approximately –1.4 MPa, corresponding to severe water deficit (Zgallaï et al., 2006; Silva et al., 2012). In WS2, 100 mL of water was applied every 4–5 days once Ψ_stem_ dropped below this threshold, preventing irreversible wilting while keeping plants under severe stress.

### 2.2 Stem water potential and stress integral

Monitoring the periods of stress and their intensity was conducted by measuring stem water potential (Ψ_stem_) at midday using a Scholander pressure chamber (PMS Instrument Company). Measurements were performed on the fifth fully expanded leaf from the shoot apex of each plant (one leaf per plant) on four plants per treatment combination, across nine sampling dates throughout the experiment. Additional information about the treatments and graphical representation of Ψ_stem_ for each treatment throughout the experiment can be found in Peco et al (Peco et al., 2023). Stress integrals (SI) were calculated from Ψ_stem_ data following Myers (1988); this index was previously employed in studies on greenhouse tomatoes by Alomari–Mheidat et al. (2024):


SI=|∑(WP−r)∗n|

Where:

- WP was the average Ψ_stem_ (MPa) between two consecutive sampling dates.
- R was the reference value obtained from the equation R = –0.018 x T_max_, with T_max_ being the daily maximum temperature (°C) on each date.
- N was the number of days between the two sampling dates.

### 2.3 Gas exchange parameters

Midday measurements of net photosynthesis (A) and substomatal CO_2_ concentration (Ci) were conducted on the fifth fully expanded leaves from the shoot apex of each plant, with one leaf selected per plant and four plants (replicated) per treatment-genotype combination per sampling date. Employing a CIRAS–3 DC CO_2_/H_2_O Gas Analyzer (PP–Systems, Amesbury, MA, USA) equipped with an automatic universal leaf cuvette (PLC6–U, PP–Systems), these assessments were performed. The gas exchange results were expressed as percentage changes relative to C (%).

### 2.4 Chlorophyll content

Leaf tissue was extracted in 80% (v/v) acetone overnight at 4°C in darkness, centrifuged (15–000 g, 5 min, 4°C) and the absorbance of the supernatant measured at 663 and 647 nm. For each genotype and treatment, one leaf per plant from four plants (replicates) was sampled, yielding eight leaf samples per sampling date. Samples were collected on four sampling dates during the experiment. Chlorophylls *a* and *b* were calculated according to Lichtenthaler and Buschmann (2001) and expressed on a dry–weight basis (mg g⁻¹ DW).

### 2.5 Chlorophyll fluorescence measurements

Chlorophyll *a* fluorescence was measured on leaves dark–adapted for at least 20 minutes using a Handy–PEA^®^ chlorophyll fluorometer (Handy–Plant Efficiency Analyser, Hansatech Instruments, King’s Lynn, Norfolk, UK). The fluorescence transients were induced by 1s illumination with an array of six light–emitting diodes, providing a maximum light intensity of 3000 µmol (photons) m^−2^ s^−1^ and uniform irradiation over a 4 mm diameter leaf area. The fast fluorescence kinetics (F_0_ to F_m_) were recorded from 10 µs to 1 s. All fluorescence measurements were performed between 11:00 and 13:00 h solar time, immediately before gas-exchange measurements, to minimize diurnal variability. Two leaves per plant were measured on eight sampling dates during the experiment, and the data were expressed as percentage changes relative to C (%).

#### 2.5.1 Analysis of the fluorescence transients using the JIP–test

Raw fluorescence OJIP transients were transferred with the PEA+ Software. This software provides a comprehensive tool for in–depth analysis of data recorded according to the equations of the JIP–test parameters by any tabulation program (Strasser et al., 2000, 2004).

The concept of the JIP–test parameters showed in this study is based on the Energy Flux Theory in Bio–membranes and the basic concept that the fluorescence yield of PSII is determined by the state open or closed of the reaction center (Strasser, 1978, 1981). The JIP–test defines the maximal (subscript ‘‘o’’) energy fluxes in the energy cascade for the events absorption (ABS), trapping (TR), electron transport (ET) and dissipation (DI) and formulated their link with selected fluorescence experimental signals (Ft) between Fo and Fm. Measured and calculated parameters are listed in **Table 1** and detailed calculation formulas can be found elsewhere (Christen et al., 2007).

**Table 1:** Parameters and explanation of the OJIP–Test parameters used in this study.

Parameters	Definition
Vitality index	
PI_ABS_	Performance Index on absorption basis integrates ABS–TR–ET–DI
Quantum efficiencies, flux ratios of PSII	
ϕ_P0_	Maximum quantum yield of primary photochemistry (Q_A_ reduction) (= TR_0_/ABS = Fv/Fm).
Ψ_E0_	Probability that a trapped exciton is used for electron transport beyond Q_A_ (= ET_0_/TR_0_).
ϕ_E0_	Quantum yield of electron transport (ABS → PQ) (= ET_0_/ABS)
ϕ_D0_	Quantum yield of non–photochemical deexcitation (heat dissipation) (DI_0_/ABS)
Phenomenological energy fluxes per excited cross section CS_0_; subscript 0 refers to time t = F_0_	
ABS/CS_0_	Absorption flux per CS
TR_0_/CS_0_	Trapped energy flux per CS (energy used to reduce Q_A_)
ET_0_/CS_0_	Electron transport flux per CS (energy reaching PQ pool)
DI_0_/CS_0_	Dissipated energy flux per CS (energy released as heat)

### 2.6 Statistical analysis

All datasets were first examined for normality (Kolmogorov–Smirnov test) and homogeneity of variances (Levene’s test). When these parametric assumptions were satisfied, analysis of variance (ANOVA) was carried out. Significant differences among treatment means were identified with Duncan’s multiple range test (p ≤ 0.05). For planned pairwise comparisons, Student’s t–test (two–tailed, p ≤ 0.05) was employed. Analyses were performed in SPSS v. 29 (IBM Corp., Armonk, NY, USA).

## 3 Results

### 3.1 Evaluation of water stress intensity through the stress integral

**Figure 1:**
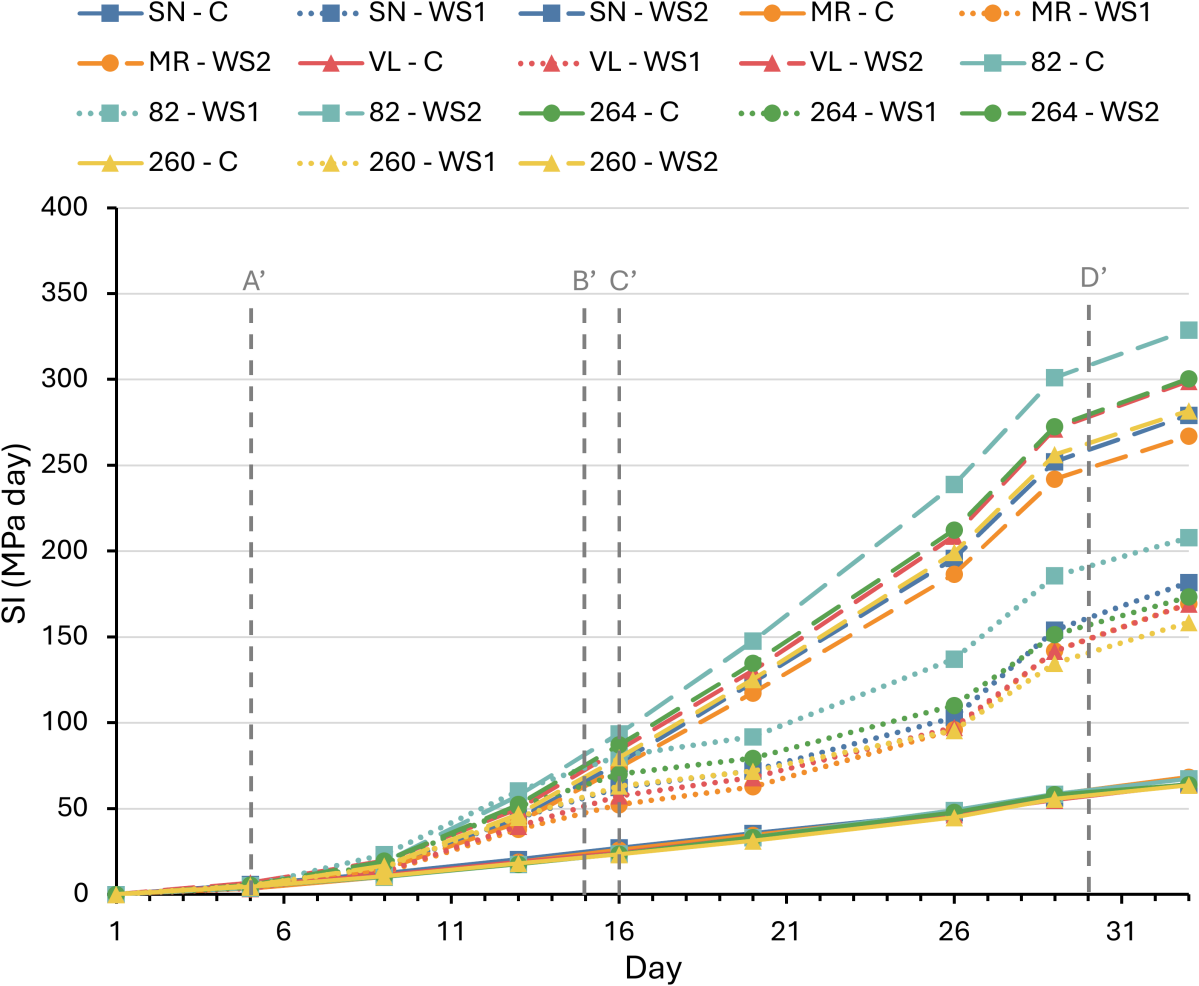
Stress integral (SI) in six tomato varieties subjected to full irrigation (C), water stress treatment 1 (WS1), and water stress treatment 2 (WS2). Vertical dashed lines indicate the following time points: A’ (onset of water stress in WS1 and WS2 plants), B’ (end of water stress in WS1 plants), C’ (onset of second water stress in WS1 plants), and D’ (end of water stress in WS1 and WS2 plants). Data represent the means of four replicates. Significant differences among treatments are summarized in **Supplementary Table S1**.

The temporal evolution of the stress integral (SI) confirmed that the three irrigation regimes produced clearly differentiated patterns (**Figure 1**). In C, SI increased slowly and almost linearly, reaching final values of 75–80 MPa · day. Under WS1, SI doubled relative to the control (180–220 MPa · day). The first drought pulse accounted for roughly 45% of the total accumulation and the second for another 40%, indicating that rewatering only partially alleviated the deficit. The prolonged stress imposed by WS2 caused a stable rise in SI, reaching maximum values of 280–340 MPa · day, almost twice that of WS1 and four times C. The slopes of the curves were virtually parallel across cultivars; however, ANOVA conducted at the points of maximum stress and after rewatering (days 13 and 16 for WS1, and days 29 and 33 for WS1 and WS2) revealed significantly greater SI values in cultivar ‘82’ at the end (**Supplementary Table S1**). Overall, these curves demonstrate that the intensity of the water deficit was dictated by the irrigation protocol and was otherwise homogeneous among the six cultivars, providing a robust framework for interpreting the comparative physiological responses discussed below.

### 3.2 Net photosynthesis, substomatal CO_2_ concentration and chlorophyll concentration

**Figure 2:**
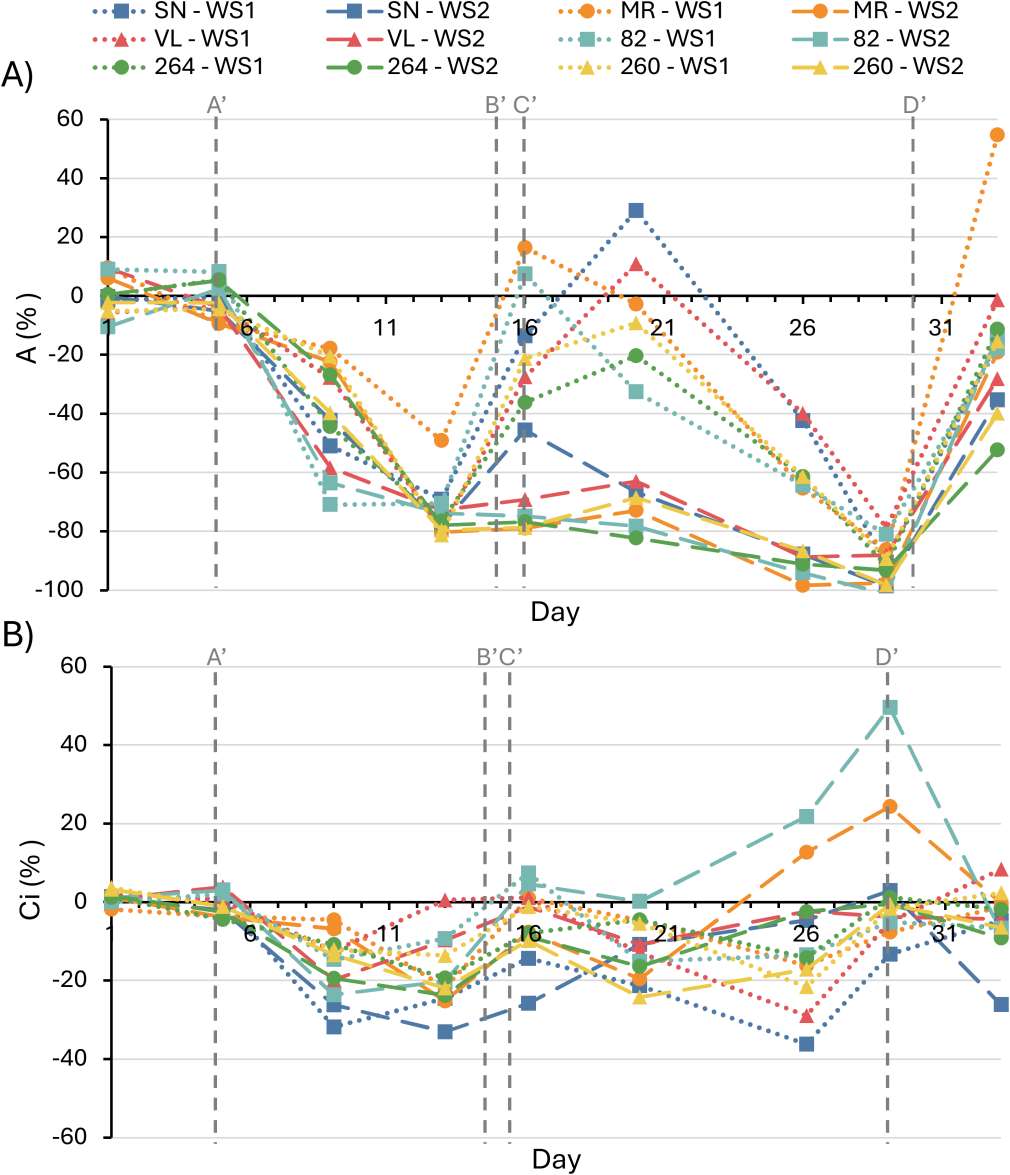
Net photosynthesis rate **(A)** and substomatal CO_2_ concentration **(B)** in six tomato varieties subjected to water–stress treatment 1 (WS1) and water–stress treatment 2 (WS2). Values are expressed as the percentage change relative to the fully irrigated control. Vertical dashed lines indicate the following time points: A’ (onset of water stress in WS1 and WS2 plants), B’ (end of water stress in WS1 plants), C’ (onset of second water stress in WS1 plants) and D’ (end of water stress in WS1 and WS2 plants). Data represents the means of four replicates. Significant differences among treatments are summarized in **Supplementary Figure S1**.

The comparison of net photosynthesis (A) values among different tomato cultivars revealed similarities in their behaviors under WS1 and WS2 treatments; however, some differences were observed in response to water stress and rehydration (**Figure 2A**; **Supplementary Figure S1**). Plants exposed to WS1 treatment exhibited a sharp decline in A from day 5, when irrigation was suppressed. On day 13, coinciding with the lowest Ψ_stem_ during the initial water stress, all plants significantly reduced their A values (–70 to –88%), with ‘MR’ showing a less pronounced decrease (–49%). Upon rehydration on day 16, all plants rapidly reached values similar to C, except for the ‘264’ cultivars, which maintained a slight decrease (–36%). Due to the second irrigation suppression in WS1 treatment, all cultivars similarly reduced their A values (–70 to –92%) corresponding to the lowest recorded Ψ_stem_ (–1.4 to –1.6 MPa). Again, the plants recovered, reaching values similar to C, except for ‘MR’, which increased its A (55%) and ‘264’, which was not able to recover the values of the control (–35%). On the other hand, all cultivars exposed to the WS2 treatment showed almost complete inhibition of A at the end of the prolonged water stress (day 29), coinciding with the lowest Ψ_stem_ (= –1.6). Once the plants were rehydrated on day 33, all cultivars regained their normal A values, except for ‘264’ (–53%) and ‘MR’ (–20%).

The results of substomatal CO_2_ concentration (Ci) in response to WS1 showed a reduction in this parameter compared to the control in the ‘SN’, 264’, and ‘260’ cultivars (–22 to –33%) during the first water stress (day 13), recovering to values similar to C during rehydration (**Figure 2B**; **Supplementary Figure S1**). During the second water stress, only ‘SN’ cultivar underwent a significant reduction (–13%), recovering to values similar to C once rehydrated (day 33). At the end of the prolonged water stress in the WS2 treatment, an increase in Ci was observed in the ‘MR’ and ‘82’ cultivars (24–49%), returning to levels similar to C once the plants were rehydrated.

**Figure 3:**
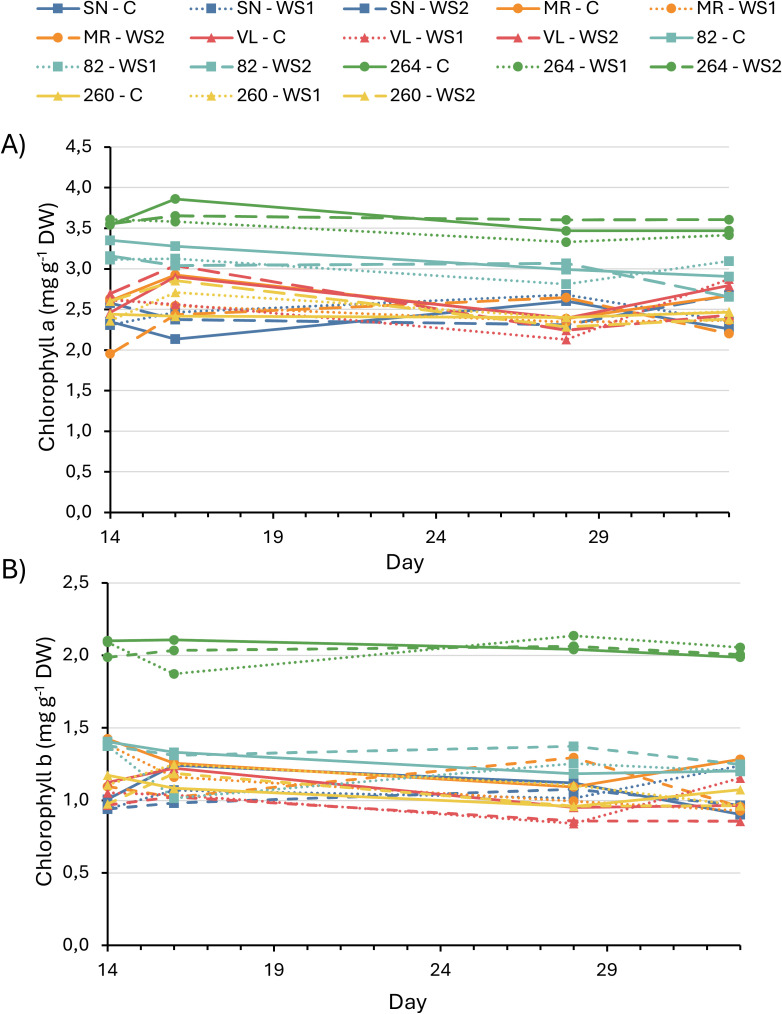
Chlorophyll a **(A)** and chlorophyll b **(B)** content in six tomato varieties subjected to full irrigation, water–stress treatment 1 (WS1) and water–stress treatment 2 (WS2). Values represent the means of four replicates. No statistically significant differences among treatments were detected within any variety (data not shown).

Chlorophyll *a* and *b* concentrations remained statistically unchanged in all cultivars and treatments (**Figure 3**), confirming that the observed physiological and photochemical adjustments were independent of pigment content.

### 3.3 Chlorophyll *a* fluorescence (OJIP) under water stress

**Figure 4:**
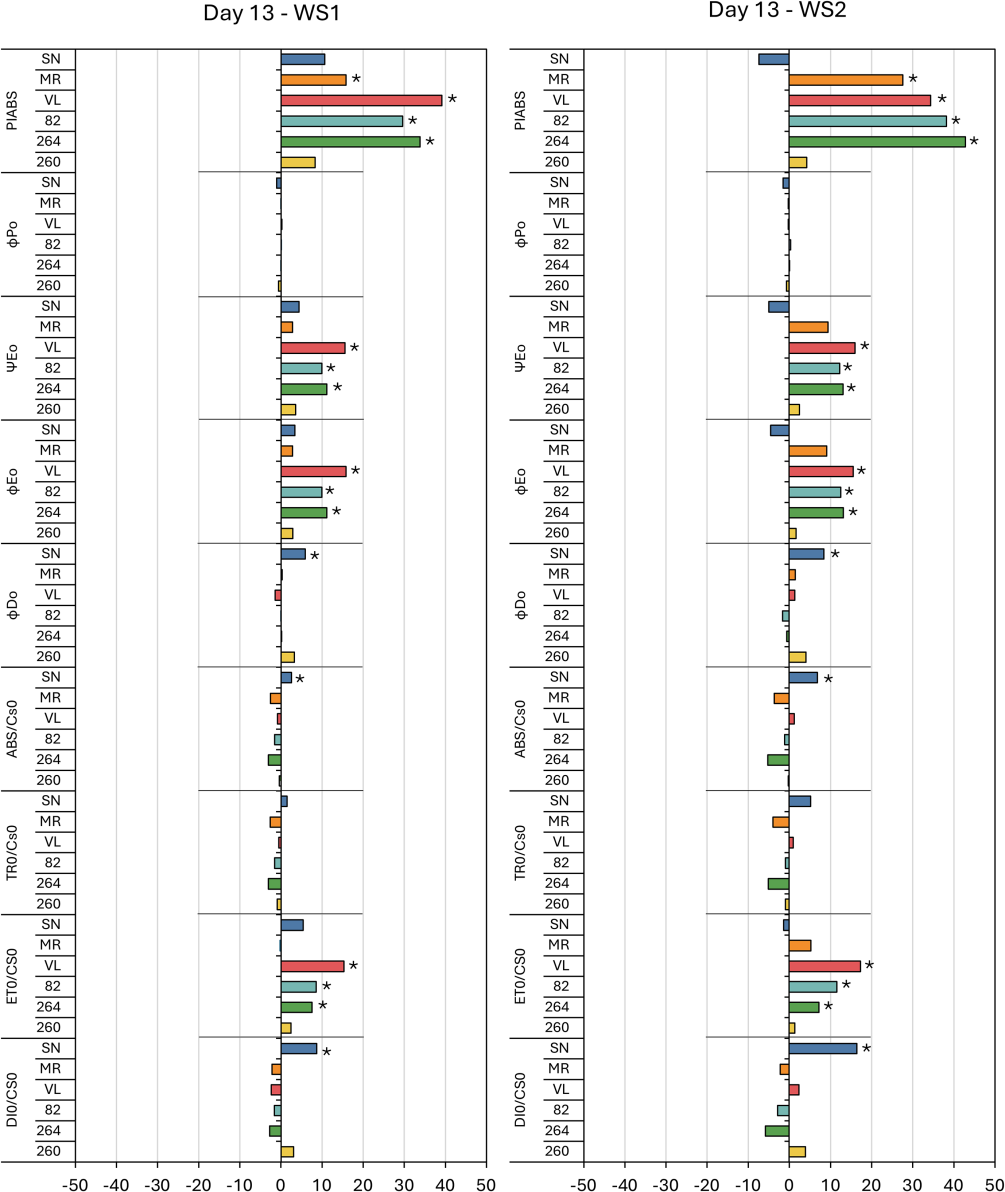
Fluorescence results during the first stress (day 13) in six tomato varieties from the water stress treatment 1 (WS1) and water stress treatment 2 (WS2). The meaning of the fluorescence parameters is summarized in **Table 1**. Values show the means of four replicates. Results were expressed in % variation with respect to control treatment. Asterisks indicate significant differences among treatments and controls at p ≤ 0.05 (t–student’s test).

**Figure 5:**
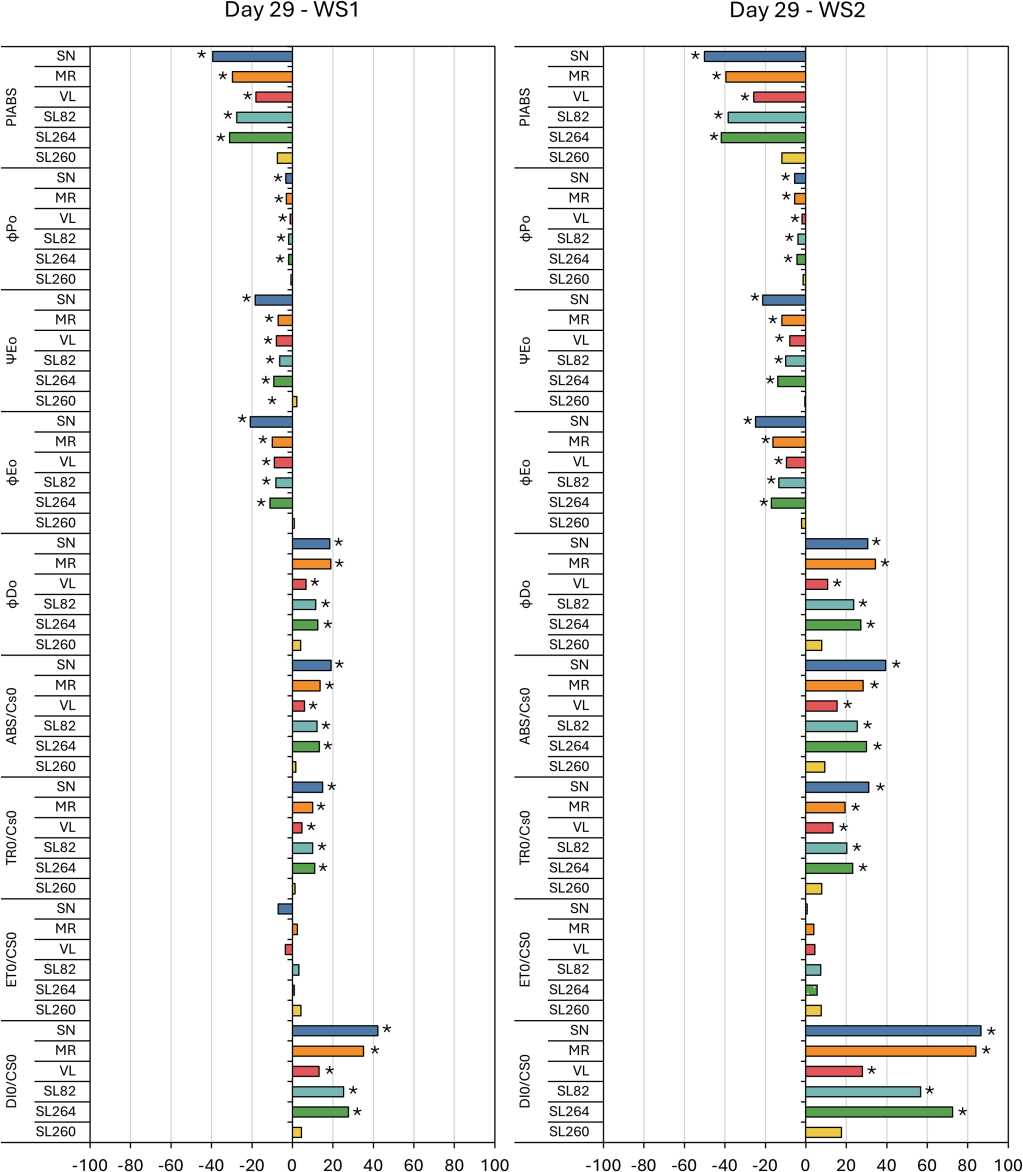
Fluorescence results during the second stress (day 29) in six tomato varieties from the water stress treatment 1 (WS1) and water stress treatment 2 (WS2). The meaning of the fluorescence parameters is summarized in **Table 1**. Values show the means of four replicates. Results were expressed in % variation with respect to control treatment. Asterisks indicate significant differences among treatments and controls at p ≤ 0.05 (t–student’s test).

**Figures 4**, **5** present the percentage variation of each JIP–test parameter in WS1 and WS2 plants relative to their irrigated C at the two stress maxima, day 13 (first drought pulse) and day 29 (cumulative stress). On day 13 the performance index on an absorption basis (PI_ABS_) increased significantly in ‘MR’, ‘VL’, ‘82’ and ‘264’, whereas ‘SN’ and ‘260’ remained unchanged; the magnitude of the increase was similar in WS1 and WS2 (**Figure 4**). By day 29, PI_ABS_ had fallen uniformly (–18% to –50%) in every cultivar except ‘260’ (**Figure 5**).

Among the energy–flux ratios, Ψ_E0_ and ϕ_E0_ differed from the control on day 13, showing moderate increases in ‘VL’, ‘82’ and ‘264’, while in ‘SN’, ϕ_D0_ also rose significantly and ϕ_P0_ unaltered (**Figure 4**). At day 29, all four ratios, ϕ_P0_, ϕ_D0_, Ψ_E0_ and ϕ_E0_, were significantly altered: ϕ_P0_, Ψ_E0_ and ϕ_E0_ decreased in every genotype except ‘260’, whereas ϕ_D0_ increased in all cultivars except ‘260’, the intensity was greater under WS2 (**Figure 5**).

Phenomenological fluxes per leaf cross–section followed a comparable pattern. Except for ‘SN’, which displayed significant increases in ABS/CS_0_ and DI_0_/CS_0_ on day 13 for both stress treatments, ABS/CS_0_, TR_0_/CS_0_ and DI_0_/CS_0_ were unaffected at the first peak but increased in all genotypes save ‘260’ at day 29, with larger increments in WS2. In contrast, ET_0_/CS_0_ rose slightly in ‘VL’, ‘82’ and ‘264’ on day 13 but showed no significant change at the second stress peak (**Figures 4**, **5**).

### 3.4 OJIP dynamics across the drought–rehydration cycles

During the first drought cycle, PI_ABS_ increased in all six cultivars (**Supplementary Figure S2**). A single re–watering of the WS1 plants was enough to return almost every value to the C level. When the second drought episode began, PI_ABS_ fell in every cultivar, most sharply in WS2. Although the final irrigation restored the index in most cultivars, ‘MR’ retained the strongest negative deviation and remained below the C for much of the monitoring period. ϕ_E0_ followed a comparable course: it rose during the initial water deficit, recovered fully after the WS1 re–watering, and increased sharply during the second stress, more markedly in WS2, with ‘MR’ again exhibiting the greatest increase. The cultivar ‘260’ was the one that underwent the least changes in both treatments.

ϕ_P0_ was virtually unchanged during the first drought, but dropped sharply under WS2 and, to a lesser extent, under WS1 in the second cycle (**Supplementary Figure S2**). Once more, ‘MR’ in WS2 was the most affected, and the final re–watering did not restore ϕ_P0_ to control values in most genotypes. Ψ_E0_ raised this parameter during the first water deficit in all cultivars except ‘MR’, which showed a slight decrease, and ‘260’, which showed the lowest. The initial re–watering (WS1) almost completely re–established Ψ_E0_, but the second drought drove it to strongly negative values, especially in WS2 and, within that treatment, in ‘MR’. In this case the final irrigation did succeed in returning Ψ_E0_ to baseline (**Supplementary Figure S3**). Thermal dissipation (ϕ_D0_) increased only modestly during the first drought (WS1) but surged in the second, particularly under WS2, and did not reach full recovery after re–watering (**Supplementary Figure S3**). The cultivar ‘260’ was the one that underwent the least changes in both treatments.

ABS/CS_0_ already rose noticeably in ‘SN’ on day 13 in both WS regimes, while in the other cultivars these fluxes showed only minor early changes but climbed steeply during the second stress (WS2 > WS1). After the final irrigation, WS1 values approached the C, whereas WS2 recovered only partially (**Supplementary Figure S3**). Capture and dissipation fluxes (TR_0_/CS_0_ and DI_0_/CS_0_) displayed the same pattern, stability in the first drought, marked rises in the second (again more pronounced in WS2), and normalization after the last irrigation in WS1, partially in WS2 (**Supplementary Figure S4**). By contrast, ET_0_/CS_0_ exhibited a more erratic course throughout the experiment and did not show a consistent trend comparable to the other parameters (**Supplementary Figure S4**).

**Supplementary Figures S5**, **S6** visualize these patterns. At day 29, WS1 and WS2 show a stressed-like OJIP shape (higher J–I and shorter I–P), with ‘260’ remaining close to the control. By day 33, the traces re-align under WS1, whereas WS2 recovery is genotype dependent.

## 4 Discussion

Water deficit is a multifactorial stress that initially restricts stomatal conductance and, if prolonged, impairs the photochemical machinery of the leaf (Flexas and Medrano, 2002; Liang et al., 2020). By integrating the three complementary data sets generated in this study: (i) the water–stress integral (SI), (ii) gas–exchange parameters (A and Ci) and (iii) OJIP chlorophyll–fluorescence kinetics, we obtained a detailed picture of how six tomato cultivars modulate these two regulatory layers under drought and how quickly they recover once irrigation is resumed. This integrative perspective reveals the dominant response mechanism in each cultivar and, by extension, its relative drought resilience.

### 4.1 Severity and uniformity of the imposed drought

The SI condenses, into a single value, both the intensity and the duration of drought experienced by a plant over a given period (Corell et al., 2022). In addition to quantifying the cumulative water status, SI serves as a proxy for drought tolerance. A high SI implies a limited capacity to reduce water loss and/or to rehydrate after each episode, traits associated with low resilience, whereas a low SI reflects early stomatal closure and/or rapid recovery, i.e., more efficient water use. In our study the SI trajectories confirmed that the irrigation protocol generated three clearly distinct water regimes (C < WS1 < WS2) and that, within each regime, all genotypes experienced comparable stress levels. This uniformity ensures that the physiological differences observed later arise from the cultivar × water–deficit interaction rather than from unequal exposure to stress. Cultivar ‘82’ accumulated significantly more SI than the other genotypes under both WS1 and WS2, pointing to a less efficient water–use strategy. That excess was corroborated by its low A under moderate drought and the sharp decline in OJIP parameters typical of drought–sensitive cultivars (Conti et al., 2019, 2021; Khan et al., 2023). The elevated SI suggests slower stomatal closure and/or slower post−stress re−hydration (Peco et al., 2023).

Hence SI, in concert with other physiological markers, could distinguish water–saving genotypes from those that sacrifice turgor, and therefore photosynthesis, during drought, providing an additional selection criterion for breeding programs aimed at improving tomato drought resilience.

### 4.2 Short term versus cumulative effects on carbon assimilation

The two drought scenarios applied, two brief pulses (WS1) versus a continuous shortage (WS2), confirm that the dynamics of A and other gas–exchange variables in tomato depend not only on stress intensity but also on drought history, as reported for pulse versus sustained deficits (Iovieno et al., 2016; Giorio et al., 2018; Peco et al., 2023).

Under drought, the primary brake on photosynthesis is usually stomatal closure, a response driven by drought, induced increases in abscisic acid that sharply reduces CO_2_ entry into the mesophyll (Xu et al., 2010; Watkins et al., 2017). If water deficit intensifies, diffusion constraints are compounded by metabolic and photochemical damage inside the chloroplast, e.g., impaired Rubisco activity, hindered RuBP regeneration and injury to PS II (Flexas and Medrano, 2002; Yuan et al., 2016). Consistent with this progression, our first WS1 pulse lowered A by 70–80% in every cultivar except ‘MR’, whose decline was limited to 49%. Re–irrigation restored A to control values within three days in all genotypes, except ‘264’, mirroring the rapid (2–3 days) recovery seen in Mediterranean tomatoes (Giorio et al., 2018) and confirming that the initial limitation was chiefly stomatal (Peco et al., 2023). The slow rebound of ‘264’, however, suggests an early metabolic impairment, consistent with the slow, often incomplete recovery typical of non–stomatal limitation (Flexas and Medrano, 2002). It is worth noting that ‘264’ showed the highest chlorophyll content among all varieties, indicating that photosynthesis and its recovery are not necessarily directly influenced by chlorophyll levels alone. A second WS1 pulse again depressed A, yet re–watering elicited a 55% over–compensation in MR, an effect reported previously in cotton (Luo et al., 2016). However, ‘264’ again failed to recover, indicating that repeated stress had shifted the dominant constraint from stomatal conductance to internal metabolism (Torrecillas et al., 2000; Flexas and Medrano, 2002; Giorio et al., 2018). During continuous drought (WS2) photosynthesis in all cultivars approached zero by day 29; after re–hydration five regained control rates, but ‘264’ retained an ≈ –50% deficit, signaling photochemical injury that exceeded the repair capacity of its photosynthetic apparatus (Flexas and Medrano, 2002; Giorio et al., 2018).

In tomato, Ci falls during mild, brief drought because stomata close restrict CO_2_ entry faster than carboxylation capacity slows. As water shortage deepens, enzymatic processes (RuBisCo activity, regeneration…) decline, reduced demand balances limited supply, so Ci returns to or even overshoots the control level (Flexas and Medrano, 2002; Cruz de Carvalho et al., 2011; Yuan et al., 2016). Under severe or prolonged stress, metabolic and photochemical damage dominate, assimilation collapses, and Ci rises well above the control (Xu et al., 2010; Yuan et al., 2016). This trajectory explains our data. In the first WS1 episode, the sharp drop in Ci recorded for ‘SN’, ‘264’ and ‘260’ denotes a predominantly stomatal limitation (Dariva et al., 2020), whereas the smaller decline in ‘MR’ and ‘82’ indicates that mesophyll demand still matched supply. Once drought became continuous (WS2), Ci in ‘MR’ and ‘82’ exceeded the control, evidencing non–stomatal constraints, that allow CO_2_ to accumulate even with almost closed stomata (Sun et al., 2016; Cardona–Ayala et al., 2020). After each re–irrigation, Ci returned to baseline in every genotype.

Taken together, these findings confirm that photosynthetic vulnerability depends on both the level and the duration of drought: short pulses induce a transient, reversible inhibition of A, whereas sustained drought provokes metabolic damage that exceeds the plant’s repair capacity, permanently shifting the A–Ψ relationship and the tolerance thresholds of each genotype.

### 4.3 Photochemical responses decoded by OJIP transients

Chlorophyll *a* fluorescence (OJIP analysis) makes it possible, with great sensitivity, to resolve the sequence of photochemical events that sustain photosynthesis. By means of integrative indices such as PI_ABS_, ϕ_P0_, Ψ_E0_, ϕ_E0_ and ϕ_D0_, one can distinguish the acclimation phases and the potential damage to the PS II apparatus under water stress.

PI_ABS_ integrates absorption, trapping, electron transport and dissipation into a single index (Chiango et al., 2021) and is therefore considered a reliable descriptor of vitality (Jedmowski and Brüggemann, 2015). In our trial, it rose transiently during the first drought pulse in ‘MR’, ‘VL’, ‘82’ and ‘264’, while it remained stable in ‘SN’ and ‘260’, a pattern already reported for *Glycine max* and *Piper nigrum* (Hlahla et al., 2022; Teles et al., 2023). This transient rise was accompanied by moderate increases in Ψ_E0_ and ϕ_E0_, together with stable values of ϕ_P0_, indicating efficient electron transfer beyond Q_A_ and improved connectivity among reaction centers (Zivcak et al., 2014; Jedmowski and Brüggemann, 2015; Suorsa, 2015). These changes suggests that moderate water deficit may enhance PSII regulatory flexibility, maintaining reaction centers (RC) operational and preventing photoinhibition (Hlahla et al., 2022; Zivcak et al., 2014; Suorsa, 2015) After prolonged drought (day 29), PI_ABS_ declined in all cultivars except ‘260’, concurrent with decreases in Ψ_E0_ and ϕ_E0,_ reflecting possible RC inactivation, over–reduction of the plastoquinone pool and exhaustion of repair mechanisms (Ghaffar et al., 2023). This late drop is the most cited response in tomato under water stress (Conti et al., 2019; Sousaraei et al., 2021). Our data therefore show that interpreting PI_ABS_ requires accounting for both the duration and intensity of the deficit, because a short drought elicits a completely different behavior from a long one.

The maximum quantum yield (ϕ_P0_ = Fv/Fm) did not change at the first water–stress point (day 13), confirming its low sensitivity to mild–moderate stress (Oukarroum et al., 2009; Mishra et al., 2012; Zivcak et al., 2014). In tomato, previous studies showed that Fv/Fm drops only when water potential falls below −1.5 MPa (Mishra et al., 2012); in wheat it fell slightly under moderate stress (Zivcak et al., 2014); and in cucumber plants it declined only after 36 days of water deficit (Teles et al., 2023). This small change in Fv/Fm is consistent with the evidence for the resistance of PS II photochemistry against moderate drought (Zivcak et al., 2014). The pronounced decrease observed during the second pulse, which did not affect ‘260’, indicates permanent PS II damage and the onset of chronic photoinhibition. Cultivar ‘260’ again behaves as drought–tolerant, as confirmed by the comparison of two tomato varieties (‘Varamin’, drought tolerant landrace, and ‘Orumie’, drought sensitive landrace), in which this index was unchanged in the tolerant variety but decreased markedly in the sensitive one (Sousaraei et al., 2021). The damage observed in the remaining varieties meant that, after re–watering, a rapid return to control values did not occur, unlike in *Vigna unguiculata*, where Fv/Fm quickly recovers and hence no serious PS II damage is inferred (Souza et al., 2004).

The biphasic behavior (decrease on day 13 and increase on day 29) of Ψ_E0_ and ϕ_E0_ matches observations in other crops showing that this marker responds differently to short versus more prolonged stress (Teles et al., 2023). ‘VL’, ‘82’ and ‘264’ displayed an initial rebound, evidence that the acceptor side (Q_B_–PQ–PS I) was still draining electrons and preventing over–reduction (Ceusters et al., 2019). A similar rise has been described in black pepper before the drought intensifies (Teles et al., 2023). After 29 days of water deficit, Ψ_E0_, ϕ_E0_ fell by –20–45% (except in ‘260’), reflecting reductive saturation of PQ and blockage after Q_A_, as seen in wheat (Zivcak et al., 2014) and *Brassica* (Antunović Dunić et al., 2023). The stability of ‘260’ points to a robust cyclic electron flow that builds up ΔpH without over–reducing the PQ pool (Lima Neto et al., 2017), again characteristic of drought–tolerant tomato lines (Sousaraei et al., 2021). All genotypes rapidly regained electron–transport values after irrigation, as previously reported in *Brassica* by Antunović Dunić et al. (2023).

The coefficient ϕ_D0_, while largely unchanged in most cultivars during the brief drought (day 13), increased significantly in ‘SN’, indicating an early activation of non–photochemical energy dissipation in this genotype. By the end of the experiment in WS1 and WS2, being highest in WS2, thus showing an accumulative–damage pattern. Nearly identical behavior has been reported in *Phalaenopsis* ‘Edessa’ (Ceusters et al., 2019) and in cowpea, although in the latter non–photochemical quenching (NPQ) soon returned to control levels (Souza et al., 2004). The late increase in ϕ_D0_ confirms that, when carbon sinks become saturated, PS II diverts excitation to non–photochemical dissipation to avoid ROS formation (Bashir et al., 2021; Peco et al., 2023). ‘260’ kept ϕ_D0_ unaltered, a trait that could be associated with fine ΔpH regulation and an efficient xanthophyll cycle (Demmig–Adams, 1990; Latowski et al., 2011; Zivcak et al., 2014), again underscoring the high drought tolerance of its photosynthetic machinery (Sousaraei et al., 2021). After rehydration it remained higher than in the control, again indicating non–reversible damage to the photosynthetic apparatus. Incomplete recovery was also observed by Antunović Dunić et al. (2023) in *Brassica* and by Chiango et al. (2021) in maize.

Finally, with the exception of ‘SN’, which displayed significant increases in ABS/CS_0_ and DI_0_/CS_0_ as early as day 13 in both water–stress treatments, the fluxes ABS/CS_0_, TR_0_/CS_0_ and DI_0_/CS_0_ did not change with short drought (day 13) but increased under prolonged stress in all cultivars except ‘260’. Similar rises in TR_0_/CS_0_ and ABS/CS_0_ have been reported in rice and grapevine (Christen et al., 2007; Guha et al., 2013). Although these increases could be attributed to thicker or more chlorophyll–rich leaves, a response sometimes triggered by drought (Yang et al., 2021; Lupa–Condo et al., 2024). In our case, chlorophyll per area did not change, suggesting RC inactivation and concentration of excitation in the remaining centers (Guha et al., 2013; Swoczyna et al., 2022). Moreover, the rise in DI_0_/CS_0_ reinforces the idea of sustained NPQ when photoassimilation is blocked, as previously discussed for ϕ_D0_.

## 5 Conclusion

The sequential changes observed across WS1 and WS2 closely fit a three-stage resilience framework in tomato. A brief water-stress pulse (WS1) triggered a reversible photoprotective adjustment, reflected in transient increases of PI_ABS_, ψ_E_0__ and ϕ_E_0__ without compromising the maximum quantum yield (ϕ_P_0__). In contrast, prolonged drought (WS2) led to cumulative photochemical damage, evidenced by sustained decreases in PI_ABS_ and ϕ_P0_, and increases in ϕ_D0_, ABS/CS_0_ and DI_0_/CS_0_, indicating reaction-centre inactivation and chronic photoinhibition. After re-watering, genotypes showed contrasting recovery capacities, confirming a genotype-dependent resilience phase. The landrace ‘260’ diverged from this pattern by keeping all OJIP parameters stable, suggesting that its resilience could rely on fine ΔpH regulation and a robust cyclic electron flow.

These findings underscore the need to interpret OJIP indices, particularly integrative ones such as PI_ABS_, considering drought duration and intensity. Assessing a single drought scenario can yield opposite conclusions: what appears as functional enhancement after short stress may mask vulnerability under sustained deficits. Including both exposure times in experimental protocols therefore allows a more precise discrimination of truly tolerant cultivars and enhances the use of fluorescence as a phenotyping tool in breeding and crop−management programs. In sum, combining the temporal dynamics of stress with the full suite of OJIP parameters provides a more realistic and predictive view of tomato photosynthetic resilience to water deficit.

## Data Availability

The raw data supporting the conclusions of this article will be made available by the authors, without undue reservation.
