# Supplementary material for: Intermittent versus continuous drought: chlorophyll a fluorescence reveals photosystem resilience in tomato
Source: Front Plant Sci. 2025 Nov 24;16:1699777. doi: 10.3389/fpls.2025.1699777 (PMC12683661; doi:10.3389/fpls.2025.1699777)
Supplement: Supplementary file 1 [file DataSheet1.pdf]

Intermittent versus continuous drought: chlorophyll *a* fluorescence reveals photosystem resilience in tomato

Peco, Jesús D.[1]; Centeno, Ana[2]; Moratiel, Rubén.[2]; Villena, Jaime[1]; López-Perales, Jesús A.[1]; Moreno, Marta M.[1,3] and Pérez–López, David [2*]

1– Dpto. Producción Vegetal y Tecnología Agraria, ETSIA–Universidad de Castilla–La Mancha, Ronda de Calatrava, 13003 Ciudad Real, Spain.

2– Dpto. Producción Agraria, CEIGRAM–Universidad Politécnica de Madrid, Av. Puerta de Hierro, 2, 28040 Madrid, Spain.

3– Instituto Botánico, Universidad de Castilla-La Mancha, Spain

***Corresponding author**: David.perezl@upm.es*

Supplementary Table S1. Significant differences of stress integral (SI) values showed in **Figure 1**. Different small letters show significant differences between treatments of all the cultivars and maximum stress points – S (13 and 29) and recovery - R (16 and 33) at P≤0.05 (ANOVA, Duncan test).

	13 (S)	16 (R)	29 (S)	33 (R)
SN-C	a	a	a	a
SN-WS1	bcde	bcd	b	bc
SN-WS2	bcde	ef	d	de
MR-C	a	a	a	a
MR-WS1	b	b	b	b
MR-WS2	bcd	def	d	d
VL-C	a	a	a	a
VL-WS1	bc	bc	b	b
VL-WS2	cdef	fg	d	ef
82-C	a	a	a	a
82-WS1	fg	ef	c	c
82-WS2	g	g	e	f
264-C	a	a	a	a
264-WS1	defg	cde	b	b
264-WS2	efg	fg	d	ef
260-C	a	a	a	a
260-WS1	bcde	bcd	b	b
260-WS2	bcde	ef	d	de

WS1	A				Ci			
	S1 (day 13)	R1 (day 16)	S2 (day 29)	R2 (day 33)	S1 (day 13)	R1 (day 16)	S2 (day 29)	R2 (day 33)
SN	⬇	↔	⬇	↔	↓	↔	↓	↔
MR	↓	↔	⬇	⬆	↔	↔	↔	↔
VL	⬇	↔	⬇	↔	↔	↔	↔	↔
82	⬇	↔	⬇	↔	↔	↔	↔	↔
264	⬇	↓	⬇	↓	↓	↔	↔	↔
260	⬇	↔	⬇	↔	↓	↔	↔	↔

WS2	A		Ci	
	S (day 29)	R (day 33)	S (day 29)	R (day 33)
SN	⬇	↔	↔	↔
MR	⬇	↓	↑	↔
VL	⬇	↔	↔	↔
82	⬇	↔	↑	↔
264	⬇	⬇	↔	↔
260	⬇	↔	↔	↔

↔ No changes

↓ Slight decrease

⬇ Moderate decrease

↑ Slight increase

⬆ Moderate increase

Supplementary Figure S1. Summary of the results of net photosynthesis (A) and substomatal CO_2 concentration (Ci) shown in **Figure 2**. Upward arrows indicate significant increases ($p \leq 0.05$) in water-stressed plants (WS) relative to the irrigated control (C), while downward arrows denote significant decreases; arrow length reflects magnitude, thin arrows represent changes < 50 %, thick arrows changes > 50 %. Sampling points: S (day 13, first stress peak), R (day 16, recovery after the first WS pulse), S (day 29, second stress peak), and R (day 33, final recovery).

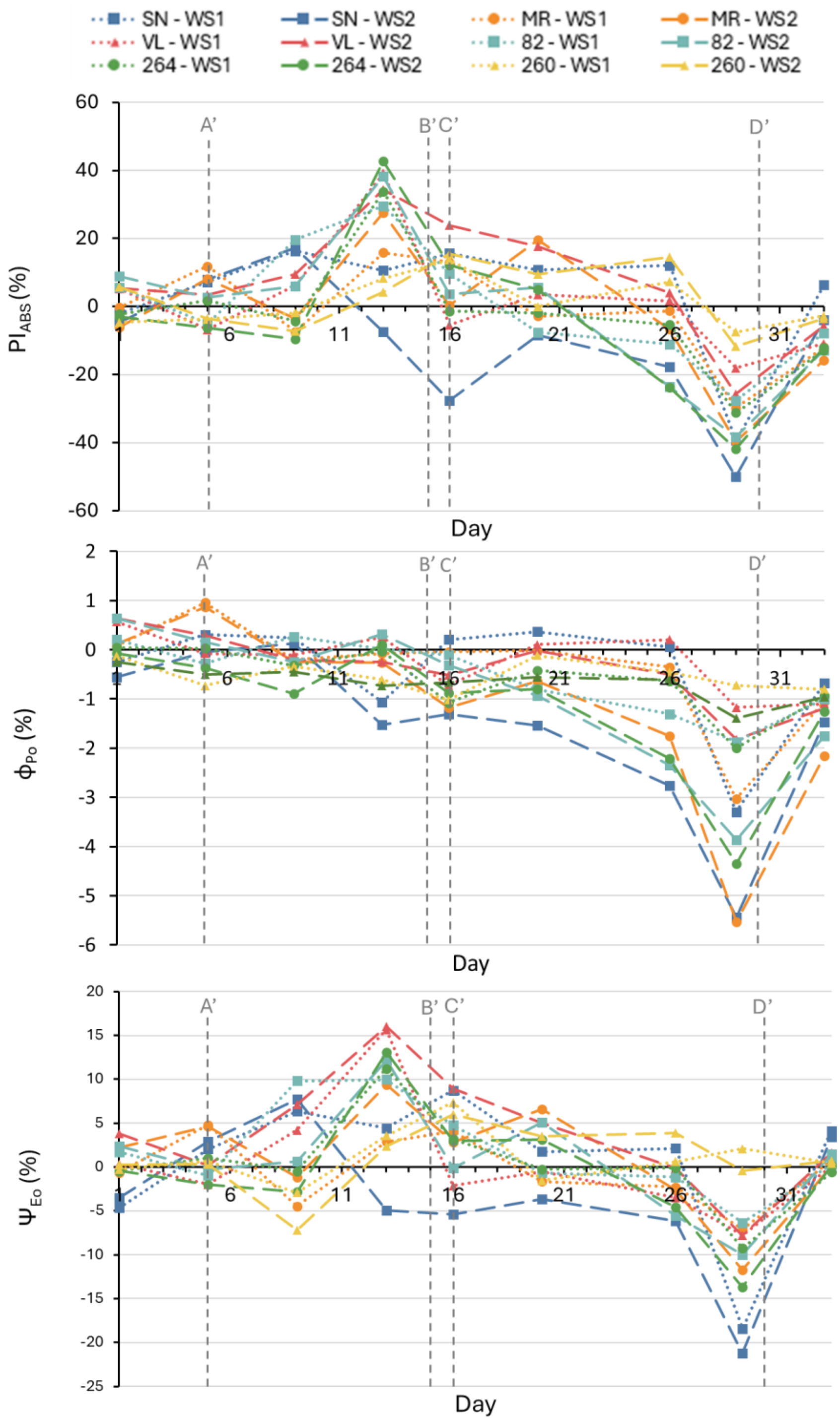

Supplementary Figure S2. Evolution of performance Index on absorption basis (PI_{ABS}), maximum quantum yield of primary photochemistry (ϕ_{P0}) and Probability that a trapped exciton is used for electron transport beyond Q_A (Ψ_{E0}) in six tomato cultivars exposed to full irrigation (C), water stress treatment 1 (WS1) and water stress treatment 2 (WS2). Vertical line A' (onset of water stress in WS1 and WS2 plants), B' (end of water stress in WS1 plants), C' (onset of second water stress in WS1 plants) and D' (end of water stress in WS1 and WS2 plants). Values show the means of four replicates. Results were expressed in % variation with respect to control treatment.

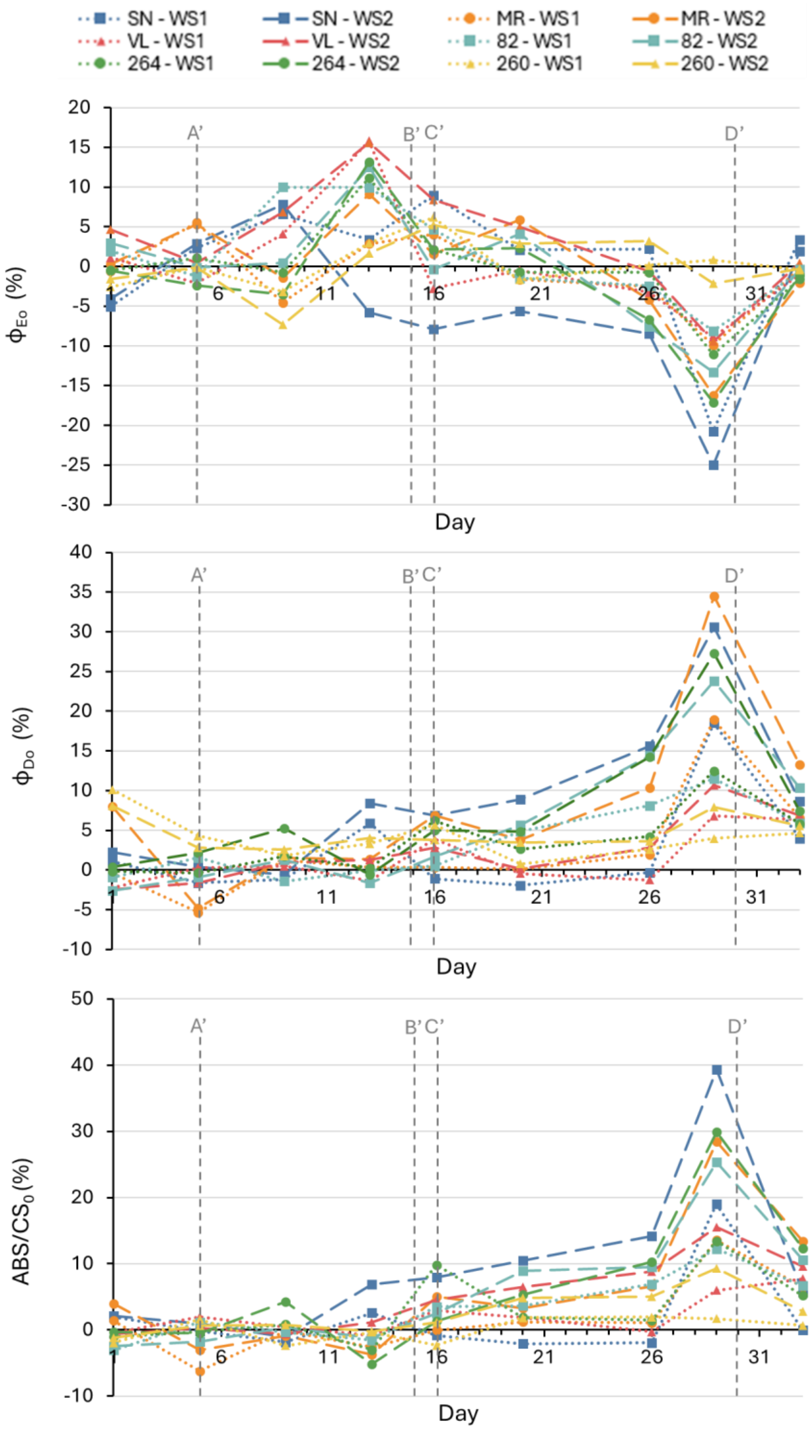

Supplementary Figure S3. Evolution of quantum yield of electron transport (ϕ_{E0}), quantum yield of non-photochemical deexcitation (ϕ_{D0}) and absorption flux per cross section (ABS/CS_0) in six tomato cultivars exposed to full irrigation (C), water stress treatment 1 (WS1) and water stress treatment 2 (WS2). Vertical line A' (onset of water stress in WS1 and WS2 plants), B' (end of water stress in WS1 plants), C' (onset of second water stress in WS1 plants) and D' (end of water stress in WS1 and WS2 plants). Values show the means of four replicates. Results were expressed in % variation with respect to control treatment.

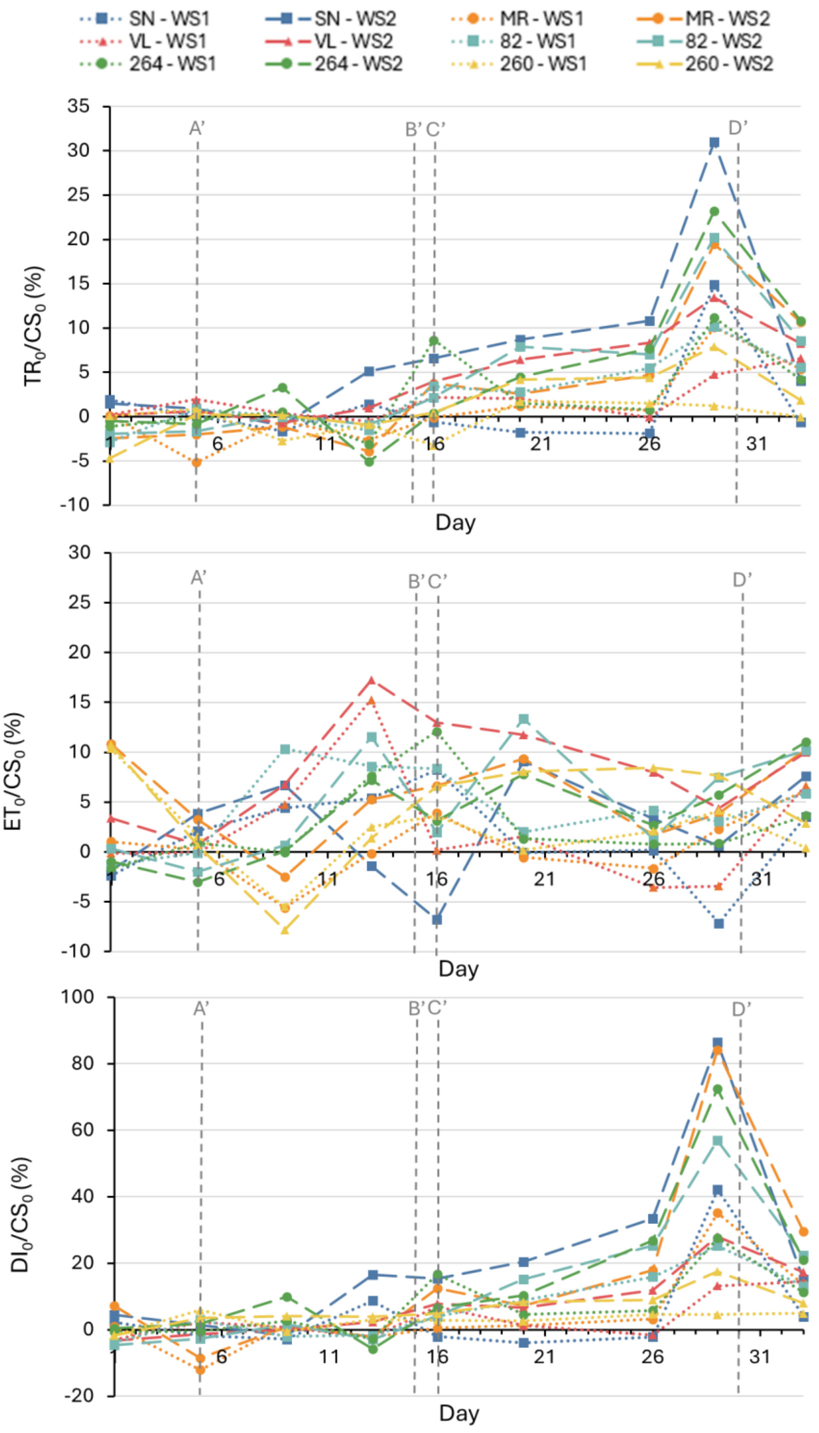

Supplementary Figure S4. Evolution of trapped energy flux per CS (TR_0/CS0), Electron transport flux per CS (ET_0/CS_0) and dissipated energy flux per CS (DI_0/CS_0) in six tomato cultivars exposed to full irrigation (C), water stress treatment 1 (WS1) and water stress treatment 2 (WS2). Vertical line A' (onset of water stress in WS1 and WS2 plants), B' (end of water stress in WS1 plants), C' (onset of second water stress in WS1 plants) and D' (end of water stress in WS1 and WS2 plants). Values show the means of four replicates. Results were expressed in % variation with respect to control treatment.

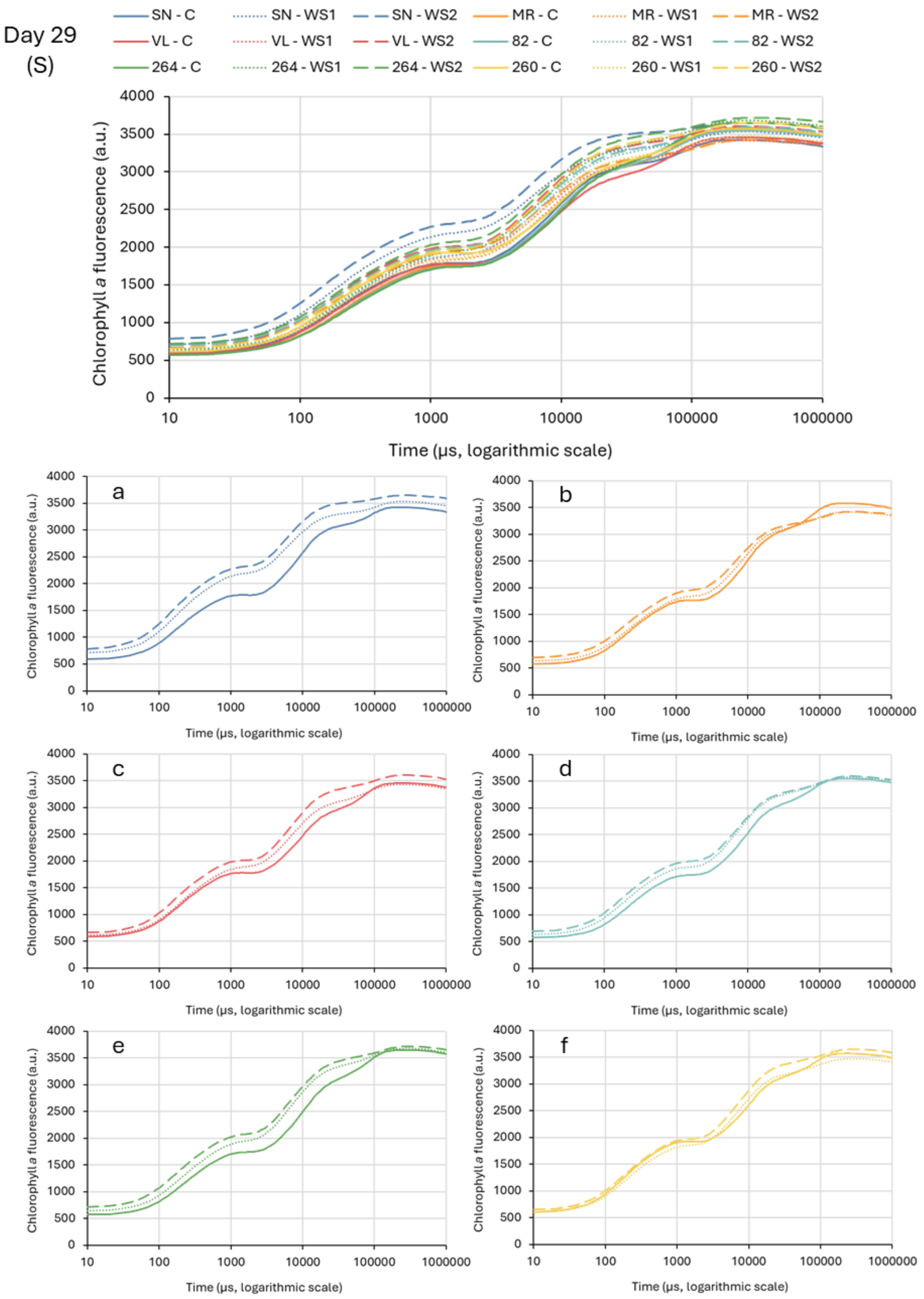

Supplementary Figure S5. Fast chlorophyll a fluorescence (OJIP) transients of tomato leaves recorded from 10 μs to 1 s at the second stress peak (day 29). The top panel displays all genotypes and treatments. Panels a–f show, for each genotype, the three treatments (Control, WS1—pulse drought, WS2—prolonged drought): Sintonía (a), Marejada (b), Valenciano (c), SL-82 (d), SL-264 (e), and SL-260 (f). Curves represent the mean of four replicates; each transient comprises 120 log-spaced sampling points.

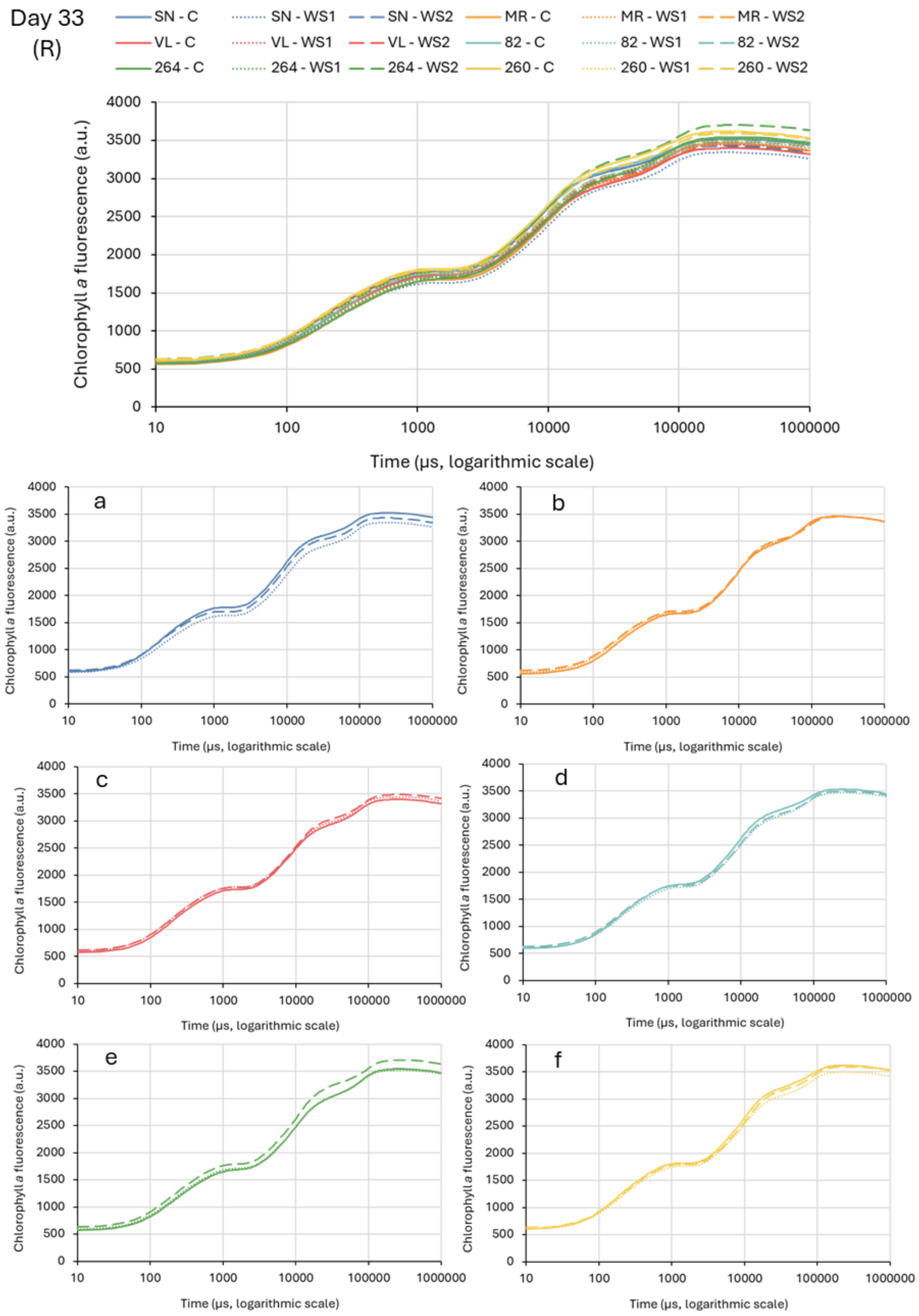

Supplementary Figure S6. Fast chlorophyll a fluorescence (OJIP) transients of tomato leaves recorded from 10 µs to 1 s at the final recovery (day 33). The top panel displays all genotypes and treatments. Panels a–f show, for each genotype, the three treatments (Control, WS1—pulse drought, WS2—prolonged drought): Sintonía (a), Marejada (b), Valenciano (c), SL-82 (d), SL-264 (e), and SL-260 (f). Curves represent the mean of four replicates; each transient comprises 120 log-spaced sampling points.
